# Red Mangrove (*Rhizophora stylosa* Griff.)—A Review of Its Botany, Phytochemistry, Pharmacological Activities, and Prospects

**DOI:** 10.3390/plants12112196

**Published:** 2023-06-01

**Authors:** Karina Kalasuba, Mia Miranti, Sri Rejeki Rahayuningsih, Wahyu Safriansyah, Rizky Riscahya Pratama Syamsuri, Kindi Farabi, Dina Oktavia, Arshad Naji Alhasnawi, Febri Doni

**Affiliations:** 1 Department of Biology, Faculty of Mathematics and Natural Sciences, Universitas Padjadjaran, Jatinangor 45363, Indonesia; karina17003@mail.unpad.ac.id (K.K.); sri.rejeki@unpad.ac.id (S.R.R.); rizky13013@mail.unpad.ac.id (R.R.P.S.); 2 Department of Chemistry, Faculty of Mathematics and Natural Sciences, Universitas Padjadjaran, Jatinangor 45363, Indonesia; wahyu17002@mail.unpad.ac.id (W.S.); kindi.farabi@unpad.ac.id (K.F.); 3 Department of Transdisciplinary, Graduate School, Universitas Padjadjaran, Bandung 40132, Indonesia; dina.oktavia@unpad.ac.id; 4 Department of Biology, College of Education for Pure Sciences, Al-Muthanna University, Samawah 66001, Iraq; arshad@mu.edu.iq

**Keywords:** *Rhizophora stylosa*, bioactive compounds, antibacterial, traditional uses

## Abstract

Mangroves are ecologically significant plants in marine habitats that inhabit the coastlines of many countries. Being a highly productive and diverse ecosystem, mangroves are rich in numerous classes of phytochemicals that are of great importance in the field of pharmaceutical industries. The red mangrove (*Rhizophora stylosa* Griff.) is a common member of the Rhizophoraceae family and the dominant species in the mangrove ecosystem of Indonesia. *R. stylosa* mangrove species are rich in alkaloids, flavonoids, phenolic acids, tannins, terpenoids, saponins, and steroids, and are widely used in traditional medicine for anti-inflammatory, antibacterial, antioxidant, and antipyretic effects. This review aims to provide a comprehensive understanding of the botanical description, phytochemical profiles, pharmacological activities, and medicinal potentials of *R. stylosa*.

## 1. Introduction

Mangroves have long been a source of interest for the public and scientists as they are extensively used for industrial and medicinal purposes [1]. They have been classified as a halophyte (salt-tolerant) that is primarily found in tropical and subtropical intertidal zones [2]. The mangrove vegetation refers to a taxonomically diverse group of trees and shrubs that dominate plant communities in tidal and salty marshes along sheltered tropical and subtropical coastlines [3]. Mangroves thrive in saline conditions with daily inundation between mean sea level and the highest astronomical tides, they provide crucial habitats and food for similarly adapted resident and migratory fauna, and they also play an important role in the ecosystem functions [4].

Due to their unique physical habitat, mangroves have evolved a set of adaptations to survive under extreme environmental conditions, such as high salinity, strong winds, gradual tidal changes, high temperature, and anaerobic tidal swamps [5]. As a direct result of this, mangroves provide a suitable habitat for a diverse array of micro- and macroorganisms that have an abundant supply of bioactive compounds and enzymes [6]. Whether it is the extensive supporting roots of *Rhizophora*, the breathing roots of *Avicennia*, the salt-excreting leaves, or the water-dispersing viviparous seedlings, everything possesses a unique capacity to produce bioactive metabolites for survival and reproduction, providing them with ‘chemical signals’ to respond to, avoid, or defend against environmental cues [2].

Mangroves have traditionally been utilized as a traditional medicinal, insecticidal, and pesticidal plant [7], as well as for fuel and charcoal, and in the construction of homes, furniture, boats, and fishing gear, as well as for the manufacturing of tannins for dyeing and leather [8]. The utilization of mangrove plants was correlated with the presence of necessary nutrients, such as amino acids, carbohydrates, and proteins, which are required for the maintenance of life processes, as well as for bioactive compounds [9].

*Rhizophora* is a type of mangrove plant that is widely distributed in the tropical Pacific region [10]. *Rhizophora* species belonging to the Rhizophoraceae family (e.g., *Rhizophora apiculata*, *R. mucronata*, *R. mangle*, *R. stylosa*), contain numerous phytochemical compounds with significant medicinal potential, such as diterpenoids, triterpenoids, sesquiterpene, daucosterol, atranorin, palmitone, polyphenols, polymeric tannins, and hydrolyzable tannins [11]. It has been reported that several *Rhizophora* species extracts possess a wide range of pharmacological activities, including antifungal, antibacterial, antiseptic, anti-inflammatory, and anti-ulcer activities [12].

*R. stylosa* Griff. is a widespread mangrove plant belonging to the Rhizophoraceae family that is widely distributed in eastern parts of the Indo-West Pacific region, from Australia to the western Pacific Ocean [13]. *R. stylosa* is one of the plants that are capable of thriving in saline environments, such as coastal areas and river estuaries [14]. *R. stylosa* is also known as the major species found in Indonesia’s mangrove ecosystem [15]. The hostile climate produces variations in the morphological, physiological, and metabolic systems of higher plant orders [2,16].

Mangroves provide distinctive biological habitats and are a rich source of bioactive compounds [17]; there are numerous compounds such as alkaloid, flavonoid, phenolic, tannin, and terpenoid saponin metabolites that can be isolated from mangrove plants [18]. Consequently, this plant is widely utilized for traditional medicine in different regions of Indonesia, including anti-inflammatory and antipyretic [19].

Although many mangrove species have been used for centuries to treat ailments in accordance with local traditions in numerous countries, many of them have not yet been subjected to extensive investigation, and their medicinal characteristics have therefore not been verified [20]. This review investigates the details of the morphological characteristics and biotechnological potentials of *R. stylosa* and its bioactive properties which can be used in pharmaceutical industries. Therefore, this work will provide a foundation for and accelerate the needed research on mangroves’ biological uses.

## 2. Methodology

An extensive literature survey of the “*Rhizophora stylosa*”, “Rhizophoraceae”, “*R. stylosa*”, “bioactivities”, “traditional uses”, “phytochemical constituents”, and “antibacterial” was conducted in scientific databases, including PubMed, Elsevier, ResearchGate, Scopus, and Google Scholar. In total, we collected over 119 publications spanning from 1984 to December 2022. There were no language restrictions imposed. All selected articles were thoroughly evaluated, and 61 publications, consisting of 60 research articles and 1 book chapter, focused on potential therapeutics, chemical components, and bioactivity of *R. stylosa* Griff. were used.

## 3. Botany

*R. stylosa* Griff. is found on sand flats or rocky sea cliffs and grows up to 15 m in height, although they are commonly much shorter, around 5–8 m, with a trunk diameter of up to 25 cm [21]. Under such conditions, trees have twisted growth, lack a distinct bole, and have vast prop roots that spiral outward. The bark of juvenile trees is reddish-brown and smooth, while the bark of adult trees is greyish-black and rough. The plant’s roots are robust even when relatively thin [10]. It has stilt roots that sprout from the trunk or branches of the mangrove and grow toward the soil, where they establish an underground root system. Stilt roots can reach a length of 3–7 m around the trunk, while aerial roots originate from the lower branches [15]. The leaf blade is widely elliptic (8–15 cm in length), the leaf blade is obovate, the base broadly cuneate, the apex has a noticeable mucronate spike, the midrib is leathery and pale green, and the underside is dotted with tiny distinct black dots. The petiole is 1–3.5 cm long, with leaflets measuring 4–6 cm. The leaf’s upper surface is glossy and smooth. Typically, the stipule is pale or yellow [15,22].

Inflorescences are axillary, branched 2–4 times with 4–8 cream-colored buds borne on the elongated peduncle [10]. The flower heads are bisexual and forked 3–5 times, with 2–too many (up to 32) flowers on a peduncle that is 1–5 cm long and attached to the axil of the leaf [15]. The floral formation consists of eight to sixteen flowers in each group. The flowers have four white corollas with dense woolly marginal hairs and a 4–5 mm style that is elongated. Flowers feature four yellow-green petals ranging in length from 13–19 mm. There are eight stamens and a 4–6 mm long pistil [10].

Long monthly day length (flowers), monthly mean air temperature (fruits), and monthly mean air temperature and relative humidity were all linked to reproductive organs (propagules). At Manko Wetland, Okinawa Island, flower buds required about 2–3 months to grow into blooms. From flower buds to fruits, the development process would take about 4–5 months. It takes about 11 to 12 months for flower buds to develop and become mature propagules, while *R. stylosa* in Peninsular Malaysia needs 10–11 months [23]. *R. stylosa*, a species with viviparous germination, adopted the hypocotyl initiation from fruit strategy. It requires about 9 to 10 months for the development of flowers into mature propagules, while the northern variety requires 11 months [24], and 11–12 months for the northeastern variety [25]. The development of fruits into mature propagules takes about 8 months [26].

The fruits are ovate and brown when ripe like the shape of water guava, and range in size from 1.5–2 cm. The hypocotyls are cylindrical, warty with a pointed tip, 2–2.5 cm in diameter, have a smooth surface, their length can reach 20–40 cm, and their collars are bulbous and yellow [27]. Flowers and fruits are produced throughout the year [15].

**Figure 1:**
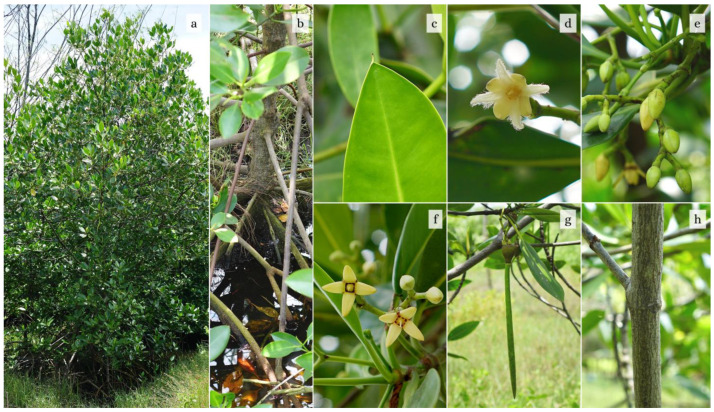
(**a**) *R. stylosa* Griff. in the mangrove forests of Karangsong Coast, Indramayu, Indonesia. (**b**) *R. stylosa* showing stilt roots. (**c**) The mucronate spike tip at the apex of *R. stylosa* leaves. (**d**) *R. stylosa* open flowers with distinctive woolly petals. (**e**) *R. stylosa* flower buds. (**f**) *R. stylosa* style. (**g**) Mature hypocotyl and fruit of *R. stylosa*. (**h**) The tree bark of *R. stylosa* (photos courtesy of Karina Kalasuba).

In the Northern Hemisphere, peak leaf litterfall for *R. stylosa* occurred from April to August, and peak stipule litterfall occurred from May to August [22,26]. The summer months have the biggest and lowest amounts of leaf and stipule litterfall, respectively. While the litterfall of fruits and blossoms peaked in July, that of propagules peaked in September. The majority of the litterfall production came from *R. stylosa*’s leaves [28,29,30]. The proportion of reproductive organ litterfall to the overall *R. stylosa* litterfall at Manko Wetland was 21.3% [26]. Figure 1 illustrates the morphological characterization of *R. stylosa*.

## 4. Phytochemistry

According to the literature review, a total of 46 compounds were able to be isolated from the leaves, flowers, fruits, stems, and twigs of *R. stylosa*. Various solvents, including n-hexane, ethyl acetate, chloroform, methanol, n-butanol, and petroleum ether, were utilized to acquire the extract for the isolation process. The desiccated sample was macerated with CHCl_3_/MeOH (1:1) at room temperature before being diluted with water and partitioned with other solvents to obtain a crude extract. Various techniques, including column chromatography on RP-18 silica gel, Sephadex LH-20, and semipreparative high-performance liquid chromatography (HPLC) on the RP-18 column, were utilized to purify the crude extract. Nuclear magnetic resonance (NMR), mass spectrophotometry, fit Fourier-transform infrared spectroscopy (FTIR), ultraviolet (UV), and polarimetry were employed to identify the compounds. Triterpenoid, steroid, lignan, megastigmadien, apocarotenoid, alanine derivates, monoterpenoid, aromatics/phenolics, flavonoid, fatty acid, and secondary alcohol groups were obtained through these chemical processes. Documentation regarding the phytochemical constituents along with their chemical class and their references is summarized in Table 1.

### 4.1. Terpenoid

**Figure 2:**
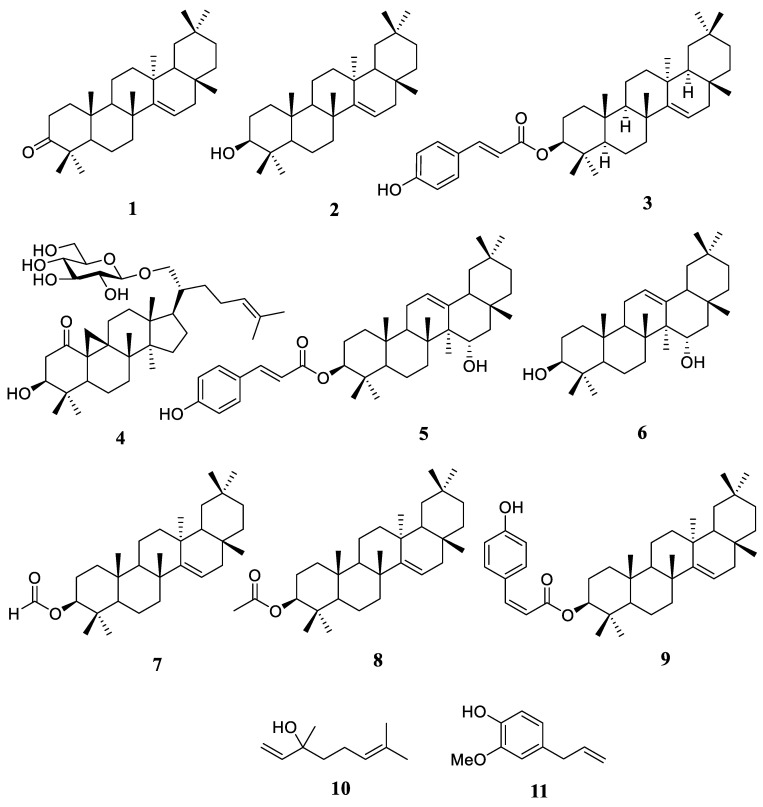
The chemical structures of compounds **1**–**11**.

A total of eleven substances were identified and characterized as triterpenoids. Taraxerone (**1**), taraxerol (**2**), and careaborin (**3**) were able to be extracted from the leaves of *R. stylosa* [31]. Subsequently, rhizostyloide (**4**) was also reported to have been identified in the leaves of *R. stylosa* [32]. Li et al. identified seven chemicals from the crude stem and twig of *R. stylosa* extract, namely 3β-*O*-(*E*)-coumaroyl-15α-hydroxy-β-amyrin (**5**), 15α-hydroxy-β-amyrin (**6**), 3β-taraxerol (**2**), 3β-taraxerol formate (**7**), 3β-taraxerol acetate (**8**), 3β-*O*-(*E*)-coumaroyl-taraxerol (**3**), and 3β-*O*-(*Z*) coumaroyl-taraxerol (**9**) [34]. Moreover, two acyclic monoterpenoid compounds have been found in the flowers of *R. stylosa*, namely linalool (**10**) and eugenol (**11**) [33], which include some essential oils. The chemical structure of compounds **1**–**11** can be seen in Figure 2.

### 4.2. Flavonoids

**Figure 3:**
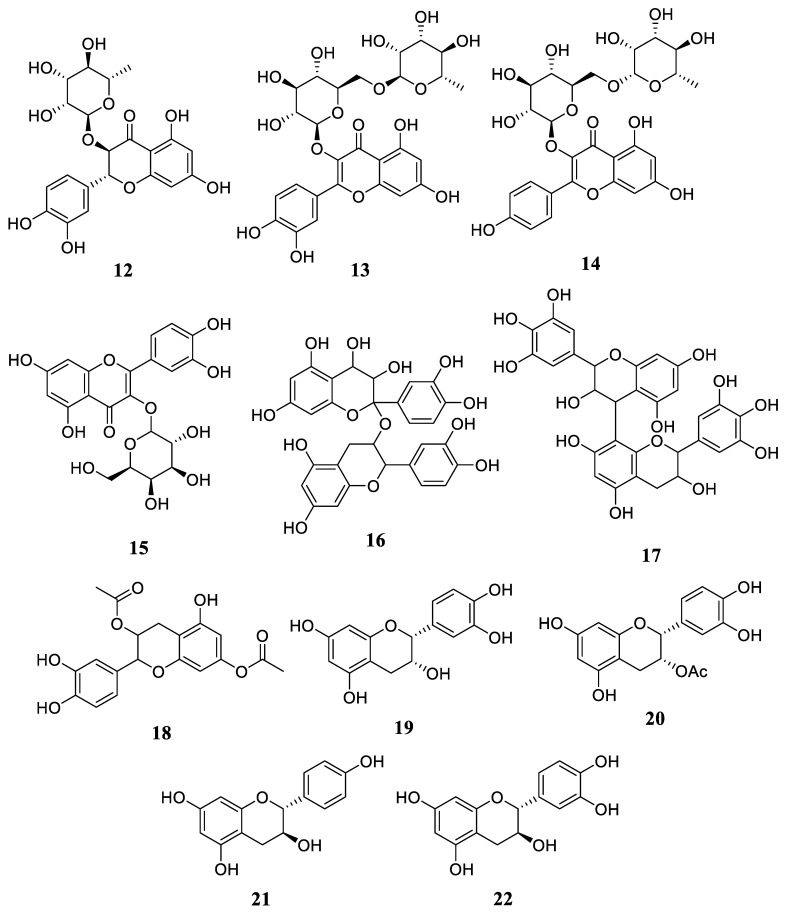
The chemical structures of compounds **12**–**22**.

**Figure 4:**
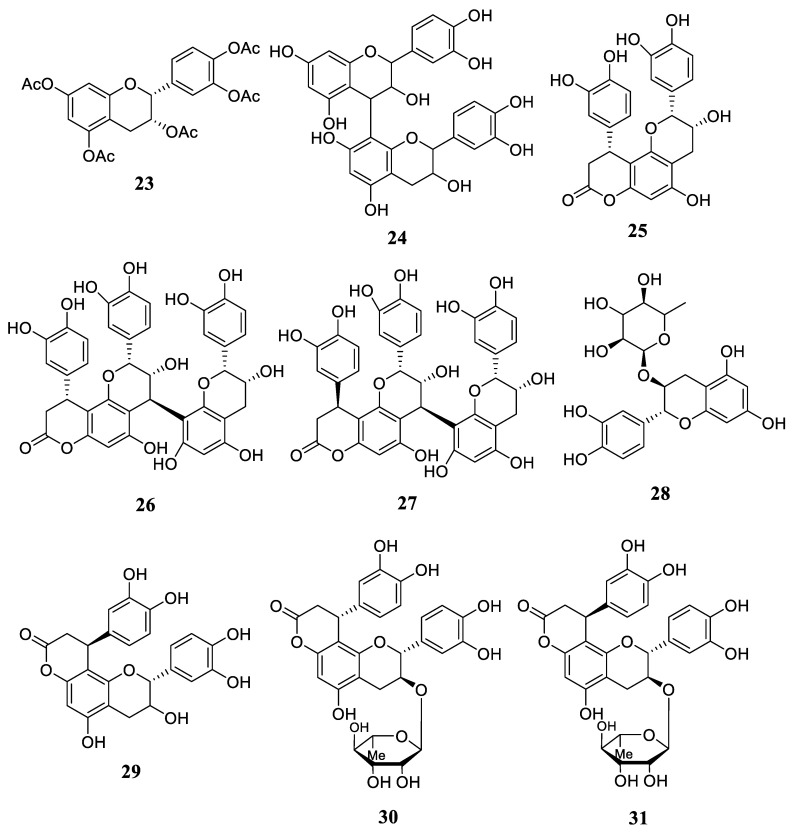
The chemical structures of compounds **23**–**31**.

Flavonoids are the most prevalent secondary metabolites discovered in *R. stylosa*. There are already twenty chemicals in this category, and Yang et al. were the first to identify astilbin (**12**) and rutin (**13**) in *R. stylosa* leaves [31]. Huong et al. also isolated kaempferol 3-rutinoside (**14**) from the leaves of the plant [32]. Miranti et al. discovered quercetin-3-*O*-galactopyranoside (**15**), procyanidin (**16**), and prodelphinidin (**17**) in *R. stylosa* fruit extracts [3], whereas the stem and twig of *R. stylosa* comprised isolated 3,7-*O*-diacetyl (−)-epicatechin (**18**), (−)-epicatechin (**19**), 3-*O*-acetyl (−)-epicatechin (**20**), (+)-afzelechin (**21**), (+)-catechin (**22**), 3,3′,4′,5,7-*O*-pentaacetyl (−)-epicatechin (**23**), and proanthocyanidin B2 (**24**) [34]. Subsequently, Takara et al. isolated seven more compounds from the stem and twig of *R. stylosa*, including cichnonain Ib (**25**), cichnonain IIa (**26**), cichonain IIb (**27**), (+)-cathecin 3-*O*-α-L-rhamnoside (**28**), cichnonain Ia (**29**), glabraoside A (**30**), and glabraoside B (**31**) [35]. The chemical structure of compounds **12**–**31** can be seen in Figure 3 and Figure 4.

### 4.3. Other Compounds

Only two steroid compounds, β-sitosterol (**32**) and β-daucosterol (**33**), have been isolated from the leaves of *R. stylosa* [31]. In addition, the leaf extract of *R. stylosa* yielded five lignan compounds, including (7*S*,8*R*)-3,3′,5-trimethoxy-4′,7-epoxy-8,5′-neolignan-4,9,9′-triol (**34**), (7*S*,8*R*)-3,3′-dimethoxy-4′,7-epoxy-8,5′-neolignan-4,9,9′-triol (**35**), (+)-isolariciresinol (**36**), polystachyol (**37**), and (+)-pinoresinol (**38**) [32].

Only one megastigmane glycoside was present and identified from the leaf extract of *R. stylosa*, specifically (6S,7E,9R)-6,9-dihydroxy-4,7-megastigmadien-3-one 9-O-[α-L-arabinopyranosyl-(l→6)-*β*-D-glucopyranoside] (**39**) [32]. In addition, Blumenol A is one of the apocarotenoid metabolites isolated and discovered from the leaf extract of *R. stylosa* (**40**) [32]. Currently, *N*,*N*-dimethyl-L-alanine is the primary alanine and its derivates have been isolated from the fruits of *R. stylosa* (**41**) [3].

**Figure 5:**
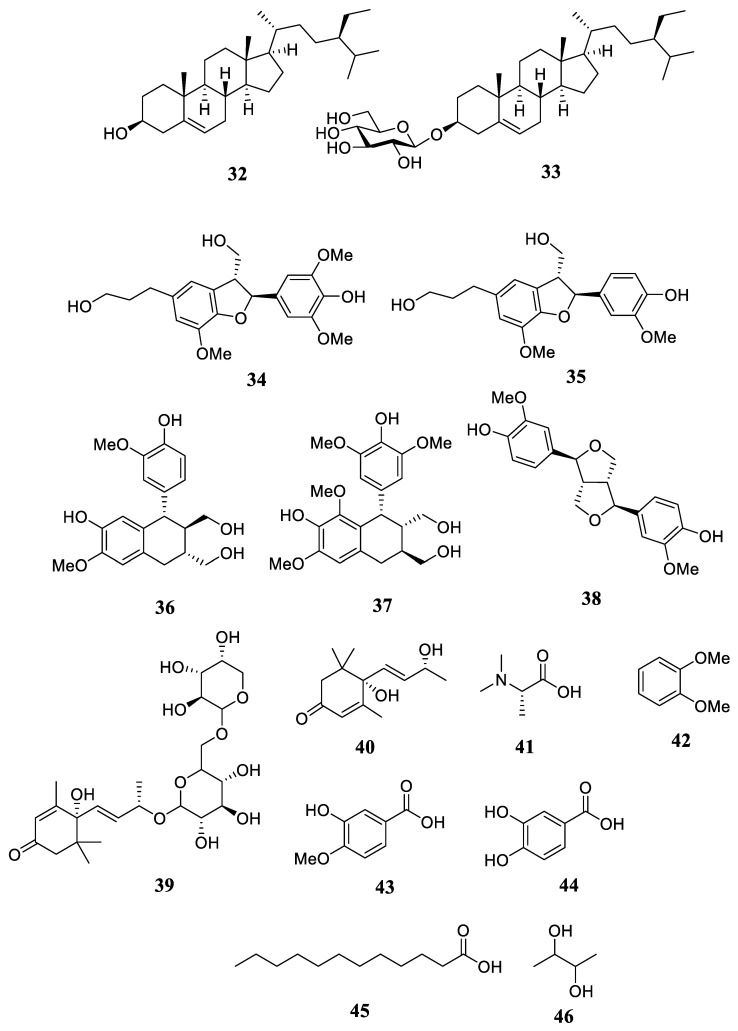
The chemical structures of compounds **32**–**46**.

Currently, only three aromatics/phenolic compounds have been found in this group, including 1,2-dimethoxybenzene (**42**) [33] from the flowers, while isovanillic acid (**43**) and protocatechuic acid (**44**) have been isolated from the leaves of *R. stylosa* [31]. Dodecanoic acid (**45**) from the fruit extract of *R. stylosa* is currently the only saturated fatty acid identified [3]. For the first time, 2,3-butanediol (**46**) was discovered in the flowers of *R. stylosa* [33]. Among similar chemicals, 2,3-butanediol was the most enticing because it is a well-known floral fragrance volatile. The chemical structure of compounds **32**–**46** is shown in Figure 5.

## 5. Ethnobotany and Medicinal Uses

Ethnopharmacological studies have also been conducted on several mangrove species. The significance of mangroves in the field of medicine for the treatment of diseases should not be neglected, as plants have a great deal of therapeutic potential. The metabolites extracted from these plants are shown to be biologically active when compared to those that become biologically active after tissue injury or pathogen invasion [36].

Traditionally, *R. stylosa* has been utilized to treat oral thrush, rheumatism, wound healing, liver disorders, and hematuria, among other conditions [37,38]. Local people in Mamuya Village and Tadupi Village, North Maluku, Indonesia have traditionally utilized the bark, young roots, and leaves of *R. stylosa* to treat a wide range of diseases; for example, by using the young roots of *R. stylosa* to treat oral thrush in infants by crushing the root in the mouth until it is smooth, followed by administering the resulting water to the infants’ mouths [37].

The leaves and stems have also been traditionally used as a remedy against liver and “lusiang” diseases, including muscle pain, back pain, bone pain, and rheumatism by mixing the leaves and stem of *R. stylosa*, boiling them, and then consuming the resulting tea twice daily. For wound healing, the fruit (propagule) of *R. stylosa* can be crushed and applied to the surface of the wound until the bleeding ceases [37]. Additionally, *R. stylosa* fruit is sometimes used to cure hematuria (blood in the urine) by fermenting the fruit with light wine [39]. Nonetheless, the mangrove plant’s pharmacological capabilities have not been scientifically verified by researchers. To shed more light on the traditional and pharmaceutical uses of these unique plants, researchers must devote more effort to the study of mangrove species, as there is a shortage of information in this field.

## 6. Biological Activities

Several *Rhizophora* spp., notably *R. stylosa*, have been used for quite a long time as traditional medicines for wound healing, rheumatism, oral thrush, hematuria, liver disorders, muscle discomfort, back pain, and bone pain. These applications have been reported in the literature, and different extracts from different parts of the plants (roots, leaves, fruits, bark) have demonstrated exciting and significant inhibitory activities in a variety of assays, including anti-diabetic [40,41], anti-cancer [31], antioxidant [3], cytotoxic [32,42], and antibacterial [20] assays, among others. Documentation regarding the bioactive potentiality of *R. stylosa* is summarized in Table 2.

### 6.1. Antibacterial Activity

Resistance of many bacteria to antibiotics is becoming increasingly prevalent, and the adverse effects associated with antibiotic use are also a significant obstacle in the treatment of infectious diseases. Hence, the discovery of novel antibacterial compounds has become an essential priority. Mangroves have been well-investigated for their antibacterial properties, as they possess highly bactericidal activities against a wide variety of pathogens. Due to the abundance of secondary metabolites in these plants, antibacterial activity has been observed in vitro [2]. The chloroform extracts of *R. stylosa* bark showed the strongest antibacterial capabilities compared to the leaf extracts, with Minimum Inhibitory Concentration (MIC) values of 0.1 mg/mL and Minimum Bactericidal Concentration (MBC) values of 6.3 mg/mL against both Gram-positive and Gram-negative bacteria, indicating that they are more efficient against certain bacteria. This impact is likely because bark extracts contain different active compounds and phytochemicals than leaf extracts [20].

In addition, *R. stylosa* leaf extracts processed with ethyl acetate had the greatest result inhibition zone, which ranged from 11 to 19 (mm) against *Escherichia coli* [43]. Since it was projected that these extracts would be effective against a variety of Gram-negative bacteria, this suggests that they might be utilized as a broad-spectrum antibiotic for the treatment of multiple bacterial diseases. In addition, Gopal et al. reported that the chloroform extract of *R. stylosa* showed the most potent antibacterial activity against *Staphylococcus aureus*, *S. epidermidis*, *S. pyogenes*, *Escherichia coli*, *Klebsiella pneumoniae*, and *Pseudomonas aeruginosa* [20].

### 6.2. Cytotoxic Activity

Cytotoxic studies are carried out to assess the toxicity of plant extracts or drugs when ingested by humans, and cytotoxic activities can also be used to evaluate the anticancer activity of plant extracts [46]. The increasing popularity of complementary and alternative medicine is primarily due to the disadvantages associated with conventional cancer chemotherapies and the presumed benefits of more natural treatment options. Yang et al. reported that the Taraxerol compound found in *R. stylosa* demonstrated cytotoxicity against HeLa and BGC-823 human cancer cells with an IC_50_ of 73.4 µmol/L, whereas the Cis-careaborin compound inhibited the growth of BGC-823 and MCF-7 human cancer cells with IC_50_ values of 45.9 and 116.0 µmol/L, respectively [31]. In addition, Rhizostyloside, a new metabolite of cyclocartane glucoside, exhibited significant cytotoxicity against three human cancer cell lines, including KB (epidermoid carcinoma), LU-1 (lung adenocarcinoma), and SK-Mel-2 (melanoma) with IC_50_ values of 86.7 and 51.0 µg/mL, respectively [32]. This number suggests that the leaf extract of *R. stylosa* is cytotoxic to HeLa cells.

### 6.3. Antioxidant Property

Free radicals or oxidative injuries caused by the inadequate reduction of molecular oxygen during aerobic respiration have a close bond with cellular damage. Normal physiological processes are maintained through the regulation of the equilibrium between the generation of reactive oxygen species by cellular activities and their elimination by the antioxidant defense system [2]. *R. stylosa* is considered a possible source of natural antioxidants due to its high concentration of phenolic compounds and flavanol derivates.

Miranti et al. evaluated the antioxidant activity of extracts, fractionated materials, and a few isolated molecules [3]. Tea mangrove extract exhibited the highest radical scavenging efficacy; consequently, it was fractionated using four distinct solvents. Acetone and methanol fractions had the highest antioxidant activity. Caffeine from tea mangrove has weak antioxidant activity (IC_50_ = 83.69 µg/mL), whereas condensed tannin (procyanidin), which is primarily found in the acetone and methanol fractions of tea mangrove extract, possesses considerable antioxidant activity (IC_50_ = 2.69 µg/mL). The condensed tannins in tea or fruit mangrove extract are primarily responsible for their antioxidant properties. Tannins are capable of donating electrons to free radicals, stabilizing them, and reducing the formation of damaging radical complexes [47]. Suh et al. reported that the methanol extract of *R. stylosa* bark had a higher phenol content and 85.5% higher antioxidant activity than aqueous extracts [45]. Li et al. also reported that proanthocyanidin, one of the isolated compounds from *R. stylosa*, had the highest antioxidant activity, with an IC_50_ of 4.3 g/mL, which is four times more active than the positive control, butyl hydroxyl toluene (BHT) (IC_50_ = 18.0 µg/mL) [34].

### 6.4. Anti-Diabetic Property

The leaves of *Rhizophora* spp. contain alkaloids, flavonoids, steroids, terpenoids, phenolic compounds, tannins, and saponins, according to the phytochemical analysis. Flavonoids are believed to have a vital role in the healing process for diabetes by enhancing the activity of antioxidant enzymes and the sensitivity of insulin receptors. Flavonoids are also known to repair injured β-pancreatic cells, allowing insulin insufficiency to be overcome; flavonoids also limit the functionality of the blood enzyme α-glucosidases. This enzyme action can effectively limit the breakdown of complex carbs and their absorption, hence lowering the rise in postprandial glucose levels in diabetics [48,49]. The ethanol extract of the leaves of mangrove *Rhizophora* spp., including *R. stylosa*, at a dose of 200 mg/kg BB + 2 g/kg glucose (group P-2), showed the highest anti-diabetic efficacy and reduced blood glucose levels by 31.27% [44]. Moreover, Efendi et al. reported that the administration of *Rhizophora* leaf extract in the treatment group was able to reduce blood glucose levels in comparison to before treatment. With a dosage of 500 mg/kg BW, there was a 29% decrease in fasting blood sugar levels [50]. These findings confirmed that *R. stylosa* possessed an anti-diabetic profile.

## 7. Prospects

Nanotechnology has become a major research breakthrough due to its extensive applications, particularly in the biomedical field [51]. One of the most studied materials is silver nanoparticles, which may be synthesized through a variety of physical, chemical, and biological processes [52]. Silver nanoparticles have immense promise in biological applications, particularly as antibacterial and antioxidant agents [53,54].

In the biological synthesis of nanoparticles, the use of phytochemical substances found in plants offers an untapped potential [55]. Biosynthesis is a plant-mediated synthesis that is more efficient than physical and chemical processes. The primary advantage of biosynthesis during nanoparticle synthesis is that no harmful chemicals, heat, energy, or high pressure are required [56].

*R. stylosa* Griff. are capable of synthesizing nanoparticles, which have recently emerged as an alternative in a variety of industries and are used for biomedical purposes [57]. In addition, the phytoconstituents extracted from mangrove plants, including gallic acid, galactose, lupeol, catechins and epicatechin, carotenoids, etc., were investigated for their diverse biological activities [58]. These compounds are utilized by the pharmaceutical and nutraceutical industries to develop antibacterial, antioxidant, anticancer, and antidiabetic medicines, among others [58]. As an antibacterial agent, it is suggested to combine silver nanoparticles (AgNPs) with a renewable source derived from mangrove plants.

Due to their small size and large surface area, silver nanoparticles with a dimension of less than 100 nm have been a major concern for researchers [59]. In a recent study by Willian et al., the antimicrobial activity of RS-AgNps was tested against two types of bacteria, i.e., *E. coli* and *Staphylococcus aureus* as representatives of Gram-negative and Gram-positive bacteria, which were commonly used to evaluate the activity of nanoparticles in earlier studies. The antibacterial testing method employed agar diffusion. Positive and negative controls consisted of amoxicillin and distilled water, respectively. The test was conducted twice, and all presented data were averaged [52].

The findings revealed that the three variations in AgNP concentration considerably affect the inhibition zone. The area of limitation for RS-AgNPs at concentrations of 0.001 M, 0.005 M, and 0.01 M is 5.5 mm, 7.2 mm, and 5.1 mm for *E. coli* and 4.3 mm for *S. aureus* bacteria. A higher zone of inhibition was seen in the 0.005 M concentration of AgNPs against *E. coli* and *S. aureus*. Willian et al. also discovered that the antibacterial activity of AgNPs synthesized with 1-, 5-, and 10 mM precursor concentrations were greater against *E. coli* than against *S. aureus* [57].

According to these studies, RS-AgNPs are more selective against Gram-negative bacteria than Gram-positive bacteria. This might be due to the different thicknesses of the cell wall of the bacteria, where the Gram-positive bacteria have a thick coating of peptidoglycan (80 nm) with covalent connections to teichoic and teichuronic acids, while Gram-negative bacteria have a thin layer of peptidoglycan (8 nm) with a lipopolysaccharide-external membrane (1–3 m) [52,57].

Another probable reason for their susceptibility to nanoparticles (NP) is that these bacteria are coated with negatively charged lipopolysaccharides. These negatively charged molecules are closer to positive ions, which are mostly released by NP, resulting in greater intracellular damage and ion absorption. NP exhibit antibacterial activity via multiple pathways, which can be summed up as a combination of reactive oxygen species (ROS) production, modulated gene expression, binding between nanoparticles and microorganism membranes through electrostatic interactions, cell wall penetration with the organelle responsible for protein synthesis, and binding metabolites, among other processes [43,60,61]. Based on these findings, it is possible to conclude that RS AgNPs have the potential to be developed as an antibacterial agent.

## 8. Conclusions

Mangrove plants have a long history of usefulness in traditional medicine and tend to be frequently utilized due to their abundance of possible natural chemical sources. Several kinds of bioactive compounds have been extracted and identified, and in vitro and in vivo studies on various metabolic activities have been conducted. In summary, *R. stylosa* extracts exhibited antioxidant, antibacterial, anticancer, and anti-diabetic effects, as well as cytotoxic activity; however, their therapeutic properties have not been confirmed. *R. stylosa* also has potential biomedical applications by utilizing a bio-friendly synthesis of silver nanoparticles (AgNPs) catalyzed by RS mangroves that can function as antibacterial agents. Thus, a deeper scientific understanding is necessary to uncover the potential of phytochemicals obtained from *R. stylosa*. Such research findings will provide novel biological compounds that can be used in the pharmaceutical industry.

## Figures and Tables

**Table 1:** Phytochemical constituents of *R. stylosa* Griff.

Phytochemical Classes	Compounds	References
Triterpenoid	taraxerone (**1**)	[31]
	taraxerol (**2**)	[13,31]
	careaborin (**3**)	[13,31]
	rhizostyloide (**4**)	[32]
	3β-*O*-(*E*)-coumaroyl-15α-hydroxy-β-amyrin (**5**)	[13]
	15α-hydroxy-β-amyrin (**6**)	[13]
	3β-taraxerol formate (**7**)	[13]
	3β-taraxerol acetate (**8**)	[13]
	3β-*O*-(*Z*) coumaroyl-taraxerol (**9**)	[13]
Monoterpenoid	linalool (**10**)	[33]
	eugenol (**11**)	[33]
Flavonoid	astilbin (**12**)	[31]
	rutin (**13**)	[31]
	kaempferol 3-rutinoside (**14**)	[32]
	quercetin-3-*O*-galactopyranoside (**15**)	[3]
	procyanidin (**16**)	[3]
	prodelphinidin (**17**)	[3]
	3,7-*O*-diacetyl (−)-epicatechin (**18**)	[34]
	(−)-epicatechin (**19**)	[34]
	3-*O*-acetyl (−)-epicatechin (**20**)	[34]
	(+)-afzelechin (**21**)	[34]
	(+)-catechin (**22**)	[34]
	3,3′,4′,5,7-*O*-pentaacetyl-(−)-epicatechin (**23**)	[34]
	proanthocyanidin B2 (**24**)	[34]
	cinchonain Ib (**25**)	[35]
	cinchonain IIa (**26**)	[35]
	cinchonain IIb (**27**)	[35]
	(+)-catechin 3-*O*-*α*-L-rhamnoside (**28**)	[35]
	cichnonain Ia (**29**)	[35]
	glabaroside A (**30**)	[35]
	glabaroside B (**31**)	[35]
Steroid	β-sitosterol (**32**)	[31]
	β-daucosterol (**33**)	[31]
Lignan	(7*S*,8*R*)-3,3′,5-trimethoxy-4′,7-epoxy-8,5′-neolignan-4,9,9′-triol (**34**)	[32]
	(7*S*,8*R*)-3,3′-dimethoxy-4′,7-epoxy-8,5′-neolignan-4,9,9′-triol (**35**)	[32]
	(+)-isolariciresinol (**36**)	[32]
	polystachyol (**37**)	[32]
	(+)-pinoresinol (**38**)	[32]
Megastigmadien	(6*S*,7*E*,9*R*)-6,9-dihydroxy-4,7-megastigmadien-3-one 9-*O*-[α-L-arabinopyranosyl-(l→6)-*β*-D-glucopyranoside] (**39**)	[32]
Apocarotenoid	Blumenol A (**40**)	[32]
Alanine derivatives	*N*,*N*-dimethyl-L-alanine (**41**)	[3]
Aromatics/Phenolic	1,2-dimethoxybenzene (**42**)	[33]
	isovanilic acid (**43**)	[31]
	protocatechuic acid (**44**)	[31]
Fatty Acid	dodecanoic acid (**45**)	[3]
Secondary Alcohol	2,3-butanediol (**46**)	[33]

**Table 2:** Bio-potency studies of *Rhizophora stylosa* Griff.

Part of Plant	Extracts Used	Phytochemical Detected	Therapeutic Application	References
Leaves	EA	Flavonoids, Alkaloids, Terpenoids, Steroids, Cardiac glycosides, and Tannin	Antibacterial	[43]
	NI	Taraxerol	Cytotoxicity	[31]
		Cis-careaborin	Cytotoxicity	
	Me	Glucoside Rhizostyloside	Cytotoxicity	[32]
	E	Alkaloids, Flavonoids, Steroids, Terpenoids, Phenolic, Tannin, and Saponins	Anti-diabetic	[44]
	Me Fractions	Procyanidin	Antioxidant	[3]
Stem and Twigs	NI	Proanthocyanidin	Antioxidant	[34]
	Me	Phenolic compounds	Antioxidant	[45]
Barks	CE	NI	Antibacterial	[20]

Notes: CE = Crude extract, E = Ethanol, NI = Not indicated, Me = Methanol, EA = Ethylacetate.

## Data Availability

This study did not report any data.
